# Competition-driven phenotypic plasticity in Iron acquisition and aromatic utilization confers a fitness advantage to *Pseudomonas putida* in an Iron-limited rhizospheric environment

**DOI:** 10.1007/s11274-024-04192-8

**Published:** 2024-11-20

**Authors:** Hiren Joshi, Atif Khan

**Affiliations:** 1 Biofouling & Biofilms Processes Section, Water & Steam Chemistry Division, BARC Facilities, IGCAR campus, Kalpakkam, 603 102 India; 2 Homi Bhabha National Institute, Mumbai, Maharashtra India

**Keywords:** Aromatic substrate, Competition, Siderophore, *Pseudomonas putida*, Rhizosphere

## Abstract

Iron scarcity poses a critical challenge for rhizospheric bacteria like *Pseudomonas putida* in the competitive rhizosphere. Despite its dependence on iron for essential functions such as root colonization, motility, and aromatic compound utilization, *P. putida* exhibits limited capability for heterologous siderophore utilization and primarily relies on the secretion of a single siderophore, pyoverdine. This study investigates the mechanisms by which *P. putida* acquires iron in an iron-limited, aromatic-rich, rhizosphere-like environment. Our findings demonstrate that *P. putida* exhibits significant phenotypic plasticity, dynamically modulating pyoverdine secretion in response to competitive pressures and substrate availability. This adaptive strategy optimizes energy expenditure and iron acquisition, providing a competitive advantage. Comparative gene expression analysis supports these observations, revealing the molecular underpinnings of this plasticity. Enhanced pyoverdine production driven by competition compensates for the bacterium's limited siderophore repertoire and facilitates rapid aromatic compound utilization, conferring a distinct fitness advantage in iron-deprived conditions. This study elucidates the complex interplay between competition, iron uptake, and aromatic compound utilization that underpins the rhizospheric success of *P. putida*.

**Supplementary Information:**

The online version contains supplementary material available at 10.1007/s11274-024-04192-8.

## Introduction

In competitive ecological niches, microorganisms are constantly engaged in life-or-death battles within diverse communities (Hibbing et al. 2010). These interactions are typically classified as (1) exploitative, involving the deprivation of essential substrates from competitors and (2) interference, where toxins and antibiotics are employed to hinder competitor growth (Cornforth and Foster 2013). Both exploitative and interference competition are closely linked to the energy status of organisms. The expenditure of energy for toxin and antibiotic secretion (interference) and the up-regulation of auxiliary pathways for quicker assimilation of available resources (exploitative) are critical determinants of competitive fitness (Cornforth and Foster 2013; Sexton et al. 2017; Maan et al. 2022; O’Brien et al. 2023). Thus, an accurate assessment of competition is essential for organisms to respond appropriately; otherwise, the metabolic efforts invested in competitor response mechanisms become futile (Maan et al. 2022). However, any delay in deploying effective strategies may result in an organism becoming outcompeted. Therefore, organisms must promptly sense competition and initiate the necessary responses (Joshi et al. 2014).

Despite its abundance, iron exhibits limited solubility at physiological pH, severely restricting its bioavailability and intensifying competition among microbes for this essential resource (Lamont et al. 2002; Hibbing et al. 2010). Siderophore secretion is a primary strategy employed by bacteria to capture environmental iron. However, this capability is widespread, with many bacteria producing siderophores, resulting in complex competitive interactions for the scarce iron supply (Niehus et al. 2017). Traits such as the ability to utilize heterologous siderophores or produce diverse siderophores confer competitive advantages, but these are largely dependent on genomic composition present in given organism (Arif et al. 2012). Interestingly, numerous organisms lacking diverse iron uptake capabilities not only survive but thrive in iron-limited environments. A comprehensive investigation into the competitive strategies employed by organisms by these organisms expected to reveal alternative iron acquisition mechanisms, thereby helping us to understand the intricate ecological dynamics within these environments.

Despite its well-documented reliance on iron for both aromatic degradation (Ramos et al. 1997) and environmental adaptation (Dinkla et al. 2001; Molina et al. 2005; Matilla et al. 2007), *Pseudomonas putida* KT2440 possesses a surprisingly limited iron acquisition repertoire. This paradox is particularly intriguing considering its competitive advantage in iron-deficient environments (Nancharaiah et al. 2008; Venkata Mohan et al. 2009; Yu et al. 2010). Notably, *P.putida* produces only a single siderophore (pyoverdine) (Matilla et al. 2007) and exhibits limited capacity to utilize heterologous ones (Matthijs et al. 2009). This apparent contradiction begs the question: how does *P. putida*, a highly competitive bacterium in aromatic-rich, iron-deficient settings, thrive with such restricted iron acquisition capabilities? This apparent contradiction fuels our current investigation into the strategies employed by *P.putida* to augment its iron supply, a critical facet we aim to unravel in this study*.*

This study investigates the mechanism(s) employed by *P.putida* KT2440 to achieve competitive success in iron-limited, aromatic-rich environments, particularly within the dynamic rhizosphere. Iron availability is critical for the root colonization and proliferation of *P.putida*, but securing this vital nutrient presents a crucial bottleneck amidst numerous competitors. This competitive landscape is further intensified by the plant's iron-deficiency response, characterized by elevated phenolic secretion, which potentially selects for bacteria capable of both iron acquisition and aromatic degradation (Shafer and Blum 1991; Jin et al. 2007, 2010). We hypothesize that *P. putida*, despite possessing only one type of siderophore (pyoverdine) and limited capability to utilize heterologous siderophore, achieves competitive success through dynamic modulation of pyoverdine secretion in response to both competition and the availability of aromatic substrates. To test this hypothesis, we evaluated siderophore secretion profiles of *P.putida* under competitive pressure in two contrasting environments: an aromatic-rich environment and environment containing an alternative substrate requiring iron-independent metabolic pathways. Our results reveal that *P.putida* dynamically modulates pyoverdine secretion in response to competition and carbon sources. This enables it to strike a crucial balance between energy conservation and effective iron acquisition, ultimately outcompeting competitors. These findings offer valuable insights into adaptive strategies adopted by *P.putida* in iron-limited environments, further supported by comparative gene expression profiling under competitive and comparative environments.

## Material and methods

### Strain and growth condition

The experiments utilized *Pseudomonas putida* KT2440 (*P.putida*), chromosomally marked with DsRed and carrying the gfpmut3b-modified KanR plasmid pWWO, as previously described (Joshi et al. 2009). Additionally, a gfpmut3b-modified Kan^R^ plasmid pWWO harboring a siderophore-negative mutant of *P.putida* KT2440 (ΔPpsD), with a mutation in the ppsD gene (PP 4219), was employed to investigate the role of siderophores in aromatics utilization. Benzyl alcohol degrading, gentamycin-resistant *Pseudomonas aeruginosa*, and *E.coli* JM101 (Non-degrader) were used for competition experiments. All the experiments were carried out in Tris minimal media { composition per liter of H2O: (A) Tris 6.05 g, Sodium chloride 4.67 g, Potassium chloride 1.5 g, Ammonium chloride 1.06 g, Sodium sulfate 0.42 g, Magnesium chloride 0.233 g, Calcium chloride 0.03 g, and Sodium dihydrogen phosphate (dehydrate) 0.004 g. (B). A trace elements stock solution: Zinc sulfate (heptahydrate) 143.77 mg/l, Magnesium chloride (Tetrahydrate) 98.96 mg/l, Boric acid 61.83 mg/l, Cobalt chloride 190.34 mg/l, Copper chloride 17.05 mg/l, Nickel chloride 23.77 mg/l, and sodium molybdate 26.29 mg/l. From this solution, 100 μl/liter was added to the Tris solution. All chemicals used were of analytical reagent (A.R.) grade and obtained from Merck, Germany} seeded with either 5 mM of Benzyl alcohol (BA) as an aromatic substrate or with 1% Glucose as the sole source of carbon and energy.

### Simulated competition experiment (for determination of absolute and relative fitness)

To demined the fitness of *P.putida*, 1X10^6^ cfu/ml of the overnight grown culture of *P.putida* (WT)/ *P.putida* (ΔPpsD) and *P.aeruginosa*/*E.coli* was inoculated in Tris- minimal media having Benzyl alcohol (5 mM) or glucose (1%) as the sole source of carbon. To determine absolute fitness, 1X10^6^ cfu/ml (equal number) of overnight grown cultures were inoculated in separate flasks. The growth of each organism was monitored by plating the appropriate dilution on selective L.B. plates (i.e., *P.putida* (WT) on Tetracycline, *P.putida* (ΔPpsD) on kanamycin, and *P. aeruginosa* on gentamicin.

For determination of relative fitness, *P.putida* (WT) or *P.putida* (ΔPpsD) were co-cultured (dual species competition in single flask) with either *P.aeruginosa* or *E.coli* in single flask, and the growth of individual cultures was monitored by plating them on to the selective plates.

### Fitness calculation

The fitness (w) of each test organism was calculated using the following formula (Bhatter et al. 2012)

1 $$\frac{{ln\left( {Df - Di} \right)}}{{ln\left( {df - di} \right)}}$$

where D*i*: Initial density (time: 0 h), D_f_: Final density (time:24 h), d_i_: Initial density(time: 0 h), d_f_ : Final density (time:24 h)

i.e., D represents the organism of interest. While d represents control, against which the fitness of the organism of interest is calculated.

### Determination of benzyl alcohol degradation/utilization by HPLC

The residual benzyl alcohol (BA) was quantified utilizing the Dionex Ultimate 3000 HPLC system equipped with an autosampler. Following centrifugation of the samples at 10,000 rpm for 2 min, 1 ml of the supernatant was transferred to the autosampler vials. The HPLC operating conditions included a mobile phase of acetonitrile: water (30:70) with 0.1% (v/v) phosphoric acid, a flow rate of 1 ml/min, a Bondapack C18 RP column, and detection at 254 nm using a UV–Visible detector.

### Determination of growth curve and siderophore (pyoverdine) production

The role of siderophores in benzyl alcohol (BA) utilization and growth was investigated by concurrently monitoring growth and pyoverdine secretion in a 96-well microtiter plate using a Biotek Synergy H5 automated multimode reader (Germany). All experiments were conducted in 150 μl of Tris minimal media supplemented with either BA or glucose as the carbon source, with a starting inoculum of 10 μl of 10^6^ cells/ml. The microtiter plates were incubated at 30 °C with intermittent shaking. Growth and pyoverdine production was monitored by measuring optical density at 600 nm and fluorescence at 395_ex_ and 455_em_, respectively, at hourly intervals using the kinetic mode of the reader. Bacterial density was calculated from the optical density vs. colony-forming units per milliliter (CFU/ml) calibration plot. The secreted pyoverdine was confirmed and quantified using the universal Chrome azurol S (CAS) assay (Schwyn and Neilands 1987).

### Per capita siderophore secretion was calculated by the following formula (Harrison et al. 2008)

2 $$A_{i} - A_{ref} / \, \ln \, \left( {Density_{i} } \right)$$

where, A_i_-: Florescence (395_ex_, 455_em_) of i^th^ sample. A_ref_ Florescence (395_ex_, 455_em_) of control. Density: cfu/ml of i^th^ sample

### Relative fitness determination of *P.putida* (WT) and *P.putida* (ΔPpsD) against multispecies natural isolates

To assess the competitive survival of *P.putida* (WT) and *P.putida* (ΔPpsD), 10^6^ cells of each respective culture were inoculated into Tris + BA media along with 1 ml of homogenized activated sludge obtained from a domestic wastewater treatment plant in Kalpakkam and microbial suspension obtained from the soil associated with the root of the plant. Daily samples were withdrawn and plated on selective media (*P.putida (*WT) on tetracycline + kanamycin and *P.putida (*ΔPpsD) on kanamycin) to determine the colony-forming units per milliliter (cfu/ml) for both cultures.

### Total RNA isolation

To obtain sufficient RNA for expression profiling, *P.putida* (10^9^ cfu/ml) was inoculated into Tris minimal media containing 5 mM of BA and grown for 1 h at 30 °C and 100 rpm. For the competition assay, 1 × 10^9^ cells of *E.coli* were co-inoculated with 1 × 10^9^ cfu/ml *P.putida* in the same flask and processed identically. After 1 h of incubation, cells were pelleted by centrifugation, and RNA synthesis was halted using RNA protect Bacteria reagent (Qiagen), following the manufacturer’s protocol. Samples were immediately processed for RNA isolation or stored at -80 °C until use.

Total RNA was isolated using the Qiagen total RNA purification kit according to the manufacturer's instructions. Purity (O.D. ratio 260/280) and concentration of isolated RNA was determined using a Tack3 plate of the Synergy H5 multiplate reader (Biotek, Germany). RNA normalization for *P.putida and P.putida* + *E.coli* was achieved by adding 2 × 10^9^ cells of *E.coli* into the *P.putida culture* already incubated for 1 h in Tris + BA media. Immediately after the addition of *E.coli*, the sample was processed for RNA isolation as described previously.

### Comparative expression profiling of genes using q-PCR

Primers used for the expression profiling are given in supplementary information. Table 1. cDNA synthesis and q-PCR were carried out using DyNAmo™ SYBR® Green 2-Step qRT-PCR Kit (Finzyme), as per instruction given by the manufacturer. RNA (100 ng) was taken for cDNA synthesis. For qPCR, 1 µl of 1:10 diluted cDNA was used for the 20 µl qPCR reaction mixture. qPCR was carried out using a Light cycler (Roche) machine, using the following PCR program: initial activation at 95** °C** for 10 min, denaturation at 95 °C for 15 s, annealing at 55 °C for 15 s, and extension with signal monitoring at 72 °C for 20 s. PCR was run for 40 cycles, followed by melting curve generation. Up/down-regulation of candidate genes was quantified using ΔΔCt methodology, using 16 s as a reference gene.

**Table 1:** Primer sequence used for Q-PCR-based expression profiling

Gene	Primer sequences	
1. ***16S***	Forward:	CCGTGTCTCAGTTCCAGT
	Reverse:	TGAGCCTAGGTCGGATTA
3. ***xylA***	Forward:	CAGCCGTTTCTGCTTACT
	Reverse:	TATCAGTCCGGCTATCGT
5. ***xylE***	Forward:	AGCATCCTCATCCACAAC
	Reverse:	GCCGTGTCTATCTGAAGG
7. ***xylR***	Forward:	CAGCCAGATCCGTTTCGTTG
	Reverse:	ATAGCCCAACCGCAGGAAGA
9. **PpsD**	Forward:	CCGCAGTACATCATCAATGG
	Reverse:	AGTGGGTTCAGGTCAACGTC
11. **FpV A**	Forward:	GCCAGAGCAGCATCTTCAAC
	Reverse:	TGACCTTGTAGCCGACCATC
7. **Xyl B**	Forward:	GATGGCTGCCGTTGTAGCA
	Reverse:	AGTTCCAGCCGGTTTTCCTT
9. **XylS**	Forward:	CGGAGCTGGCGATGATG
	Reverse:	TGGTGCCGGCATGCTT
11. **Xyl U**	Forward:	TTGGTTGCTTTCGCCATGTA
	Reverse:	TCACAGACTCCAGGCGTAACG
13. **Xyl X**	Forward:	TCGTCAATGCCTGCAGTCA
	Reverse:	GCCTTGTTTCCACTCCTAAAGC
12. **Ben R**	Forward:	CATCACGCCCAAGCATTAC
	Reverse:	AGTGGGTTCAGGTCAACGTC
14. **Ben A**	Forward:	GATTCGACTACCTCAATGCC
	Reverse:	CTCGAAGATGTGTTTCATCTCC

## Results

In natural environments, competition for limited resources shapes the coexistence of diverse species, driving each organism to optimize energy allocation and employ effective competitive strategies (Tilman, 2004). *Pseudomonas putida* KT2440 relies heavily on its iron-chelating siderophore, pyoverdine, in the rhizospheric environment, for effective iron uptake and subsequent aromatic degradation. To determine the role of siderophore in competitive fitness of *P.putida*, we conducted competition assays to compare the comparative fitness of *P.putida* (WT) and its siderophore-negative mutant lacking the pyoverdine synthase gene (ΔPpsD).

### Evaluation of competitive fitness

**Fig. 1:**
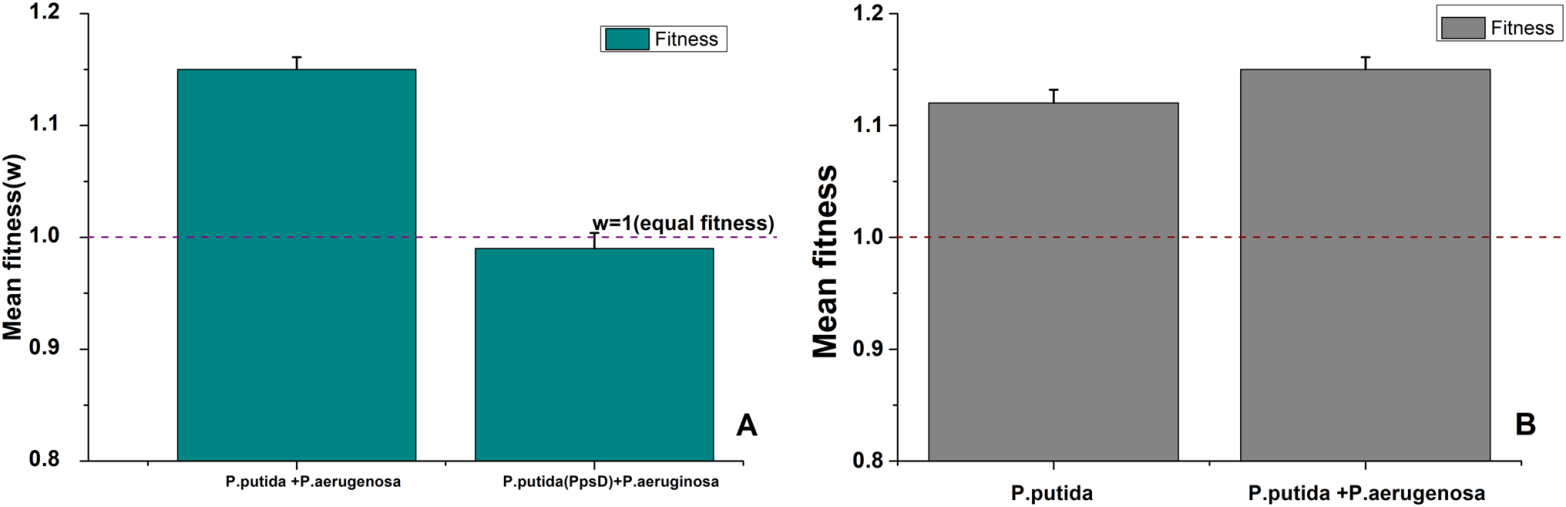
Competitive fitness of *P.putida* wild type and its siderophore mutant *P.putida* (ΔPpsD): A. Comparative fitness of * P.putida * (WT) and P.putida (ΔPpsD) against Benzyl alcohol degrading * Pseudomonas aeruginosa*. B. Absolute {*P.putida* (WT) grown as single culture) and relative {*P.putida* (WT) grown as co-culture in single flask} fitness of *P.putida * grown on Benzyl Alcohol as the sole source of carbon and energy

To initiate our investigation, we conducted a comparative fitness assessment of *P.putida (*WT) and its siderophore-negative mutant *P.putida (*ΔPpsD) against a potential competitor, *P. aeruginosa,* having the capability to utilize benzyl alcohol (BA) as the sole source of carbon and energy. Figure 1A^1^ Illustrates that *P.putida (*WT) exhibited superior fitness over the competitor compared to its mutant counterpart {fitness w = 1.12 for *P.putida (*WT) and w = 0.99 for *P.putida (*ΔPpsD)} in Tris minimal media supplemented with benzyl alcohol. Notably, when we compared the absolute fitness of *P.putida* (WT) (grown alone) and relative fitness {grown with *P. aeruginosa* in a single flask (Co-culture)}, there was better relative fitness than absolute fitness (absolute fitness 1.12, comparative fitness = 1.15) (Fig. 1B).^2^ This observation suggests a competitor effect on the growth of P.putida, wherein challenged P.putida appears to accelerate its growth in response to the presence of a competitor (resource competition). As benzyl alcohol serves as the sole source of carbon and energy, the competitor-driven growth enhancement implies an acceleration in substrate (BA) utilization. Therefore, we further investigated the rate of BA utilization by P.putida under challenged and non-challenged conditions.

### Determination of the influence of competitor on benzyl alcohol utilization by *P.putida*

**Fig. 2:**
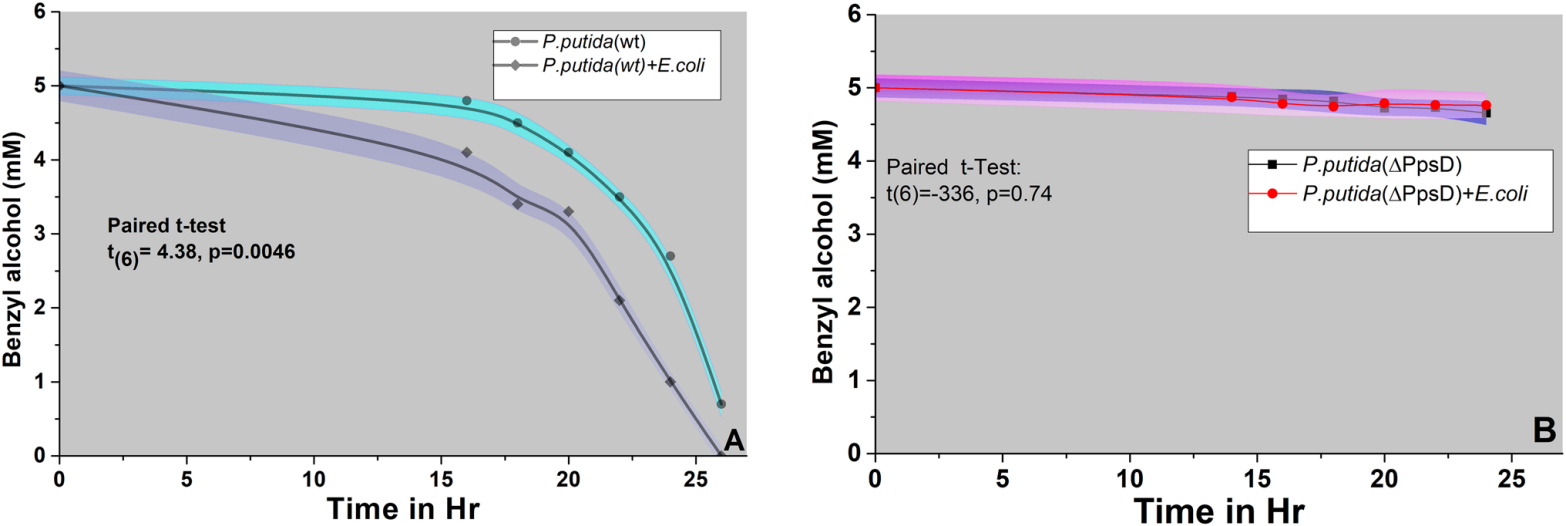
Competition and aromatic utilization: Influence of competition on Benzyl Alcohol (BA) utilization by A) *P.putida*(WT) and B) *P.putida* (ΔPpsD)

To mitigate any potential under/overestimation of aromatics degradation and siderophore secretion (explained in the next section), we opted for *E.coli*, a non-BA degrader, instead of *Pseudomonas aeruginosa*, for further experimentation. As illustrated inFig. 2A,^3^ there was a significant increase in benzyl alcohol (BA) utilization by *P.putida* (WT) in the presence of the competitor (Paired t-Test, p = 0046). However, the competitor's Influence on enhanced aromatic substrate utilization was not observed in the case of the siderophore-negative *P.putida* (ΔPpsD) (Paired t-Test, p = 0.74) (Fig. 2B).^4^ This observation, coupled with the previously established central role of siderophores in aromatics utilization (Joshi et al. 2014), clearly indicates the pivotal role of siderophores in enhanced aromatic utilization.

### Determination of growth and siderophore (pyoverdine) production

In the comparative assessment of fitness and aromatic utilization in the presence of a competitor, it is evident that *P.putida (*WT) exhibits better fitness than its siderophore mutant. However, this enhanced growth is due to higher siderophore secretion or other factors that need to be ascertained. Therefore, we have evaluated the per capita siderophore secretion by *P.putida* in a competitive and non-competitive environment. Calculating the Mean per capita siderophore will normalize the growth (number of cells) and give a clear picture of siderophore secretion by individual cells. For this experiment, we have used *E.coli* in place of P*. aeruginosa* as a competitor due to overlapping optical and chemical properties of siderophore secreted by *P.putida* and *P. aeruginosa* (Figure S1).^5^ Moreover, previously, we reported that *P.putida* shows generalized competition sensing and does not rely specifically on any particular organism (Joshi et al., 2009). Therefore, *E.coli*, a non-aromatic degrader with no overlapping optical spectra of their siderophore, was chosen as a competitor.

**Fig. 3:**
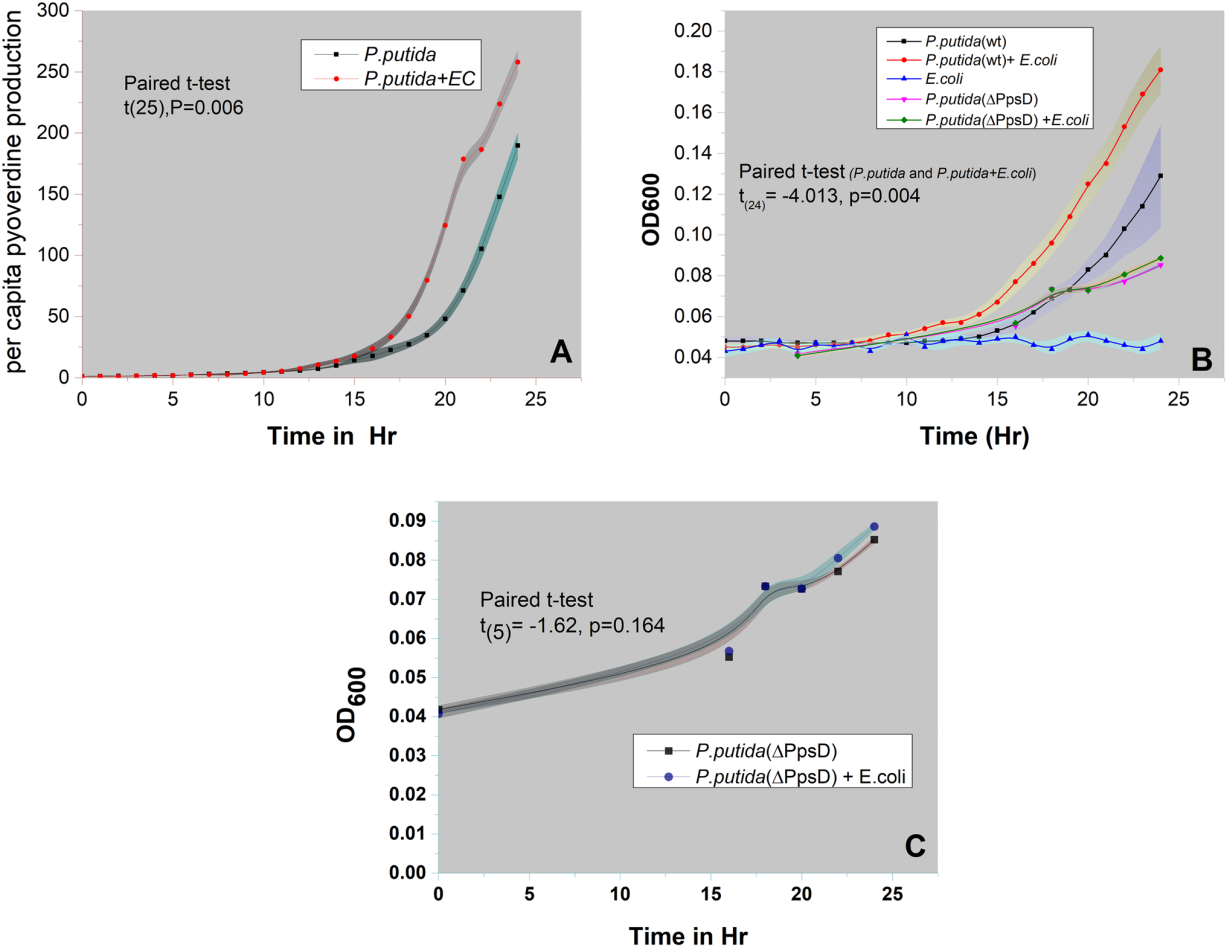
Competition-driven enhanced siderophore secretion and growth: A. Mean Per capita siderophore production by challenged and non-challenged *P.putida* (WT). B. Influence of competition on growth of *P.putida* (WT) and its siderophore negative mutant *P.putida* (ΔPpsD)

As shown in Fig. 3A,^6^ there was a significantly higher mean per capita siderophore secretion by *P.putida* when challenged with competitor compared to the non-challenged counterpart ( t-test, p = 0.006). Similarly, there was a marked increase in the growth of *P.putida* when challenged with a competitor (t-test p = 0.004) (Fig. 3B).^7^ Conversely, such competition-driven growth enhancement was not observed in the PpsD mutant (t-test, p = 0.164) (Fig. 3C),^8^ clearly establishing the involvement of siderophores in enhancing BA utilization in challenged *P.putida.* These results align with our previous report demonstrating accelerated BA utilization by *P.putida* in the presence of a potential bacterial competitor (Joshi et al. 2009).

### Influence of substrate over siderophore driven fitness advantage to *P.putida*

The utilization of aromatics by *P.putida* was demonstrated to be linked with the availability of iron, given its crucial role in the functioning of iron-dependent oxygenases responsible for the ring-opening pathway, particularly the key enzyme Catechol 2,3-dioxygenase (C23O) (Supplementary figure S2).^9^ Therefore, upregulating siderophore secretion in response to competition for aromatic substrates makes sense and provides a distinct advantage to *P.putida*. However, as siderophore secretion is an energy-consuming process, its unnecessary secretion may lead to a competitive disadvantage. Hence, it is crucial to understand how *P.putida* reacts to competitors when competing for an alternate substrate that does not require excess iron. To investigate this, we examined growth and siderophore secretion in the presence of a competitor when both were competing for an alternative substrate (glucose) as the carbon source, which does not necessitate a large amount of iron for its metabolism compared to benzyl alcohol (BA) metabolism. As illustrated inFig. 4,^10^ there was no significant difference in siderophore production under the influence of competition (*E.coli* as the competitor). To support this observation on the role of siderophore in aromatic utilization, there was no difference in mean fitness when we calculated the absolute mean fitness of *P.putida* (WT) and *P.putida* (ΔPpsD) grown on glucose (Figure S3).^11^

**Fig. 4:**
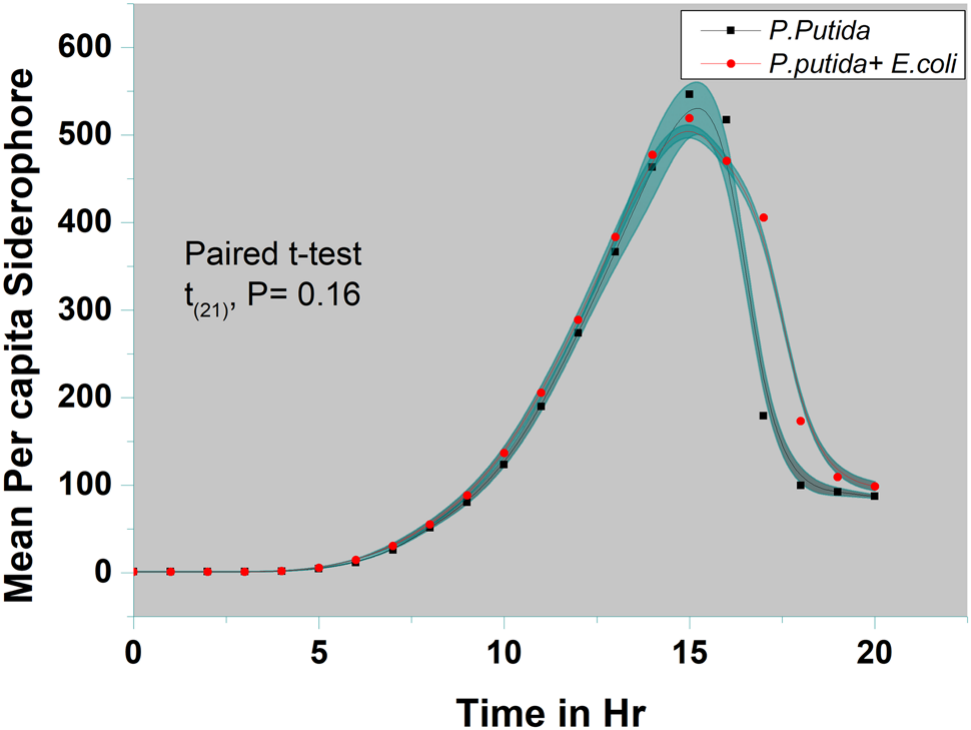
Substrate influence on competition and siderophore secretion: Influence of substrate on competition-driven siderophore secretion by *P.putida* (WT)

Reconfirming the central role of siderophore in aromatic utilization. This observation indicates the possibility of another layer of regulatory mechanism for siderophore secretion in response to the metabolic requirement of iron, which is not influenced by competition for iron-independent substrate utilization. This additional layer of regulation allows fine-tuning of siderophore secretion, preventing inefficient energy spending that could directly impact fitness and, consequently, survival.

### Comparative survival fitness against multispecies consortia (activated sludge and rhizospheric isolates)

**Fig. 5:**
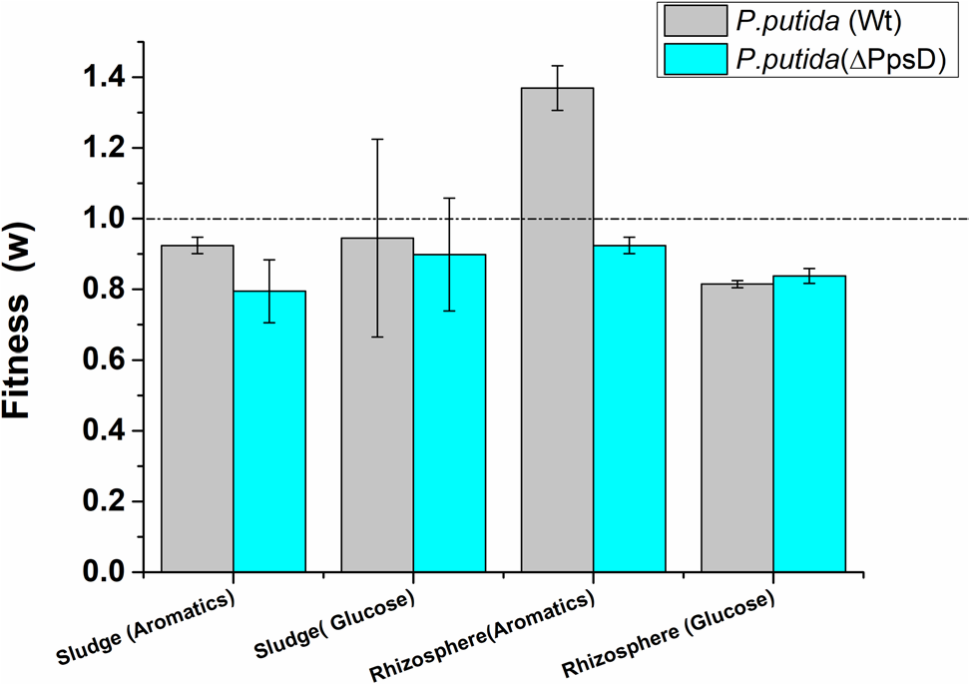
Competition in the simulated aromatic-rich environment: Comparative fitness of *P.putida* (WT) and *P.putida* (ΔPpsD) against organisms of activated sludge and rhizosphere in simulated aromatic-rich environment

Beyond its competitive success in the rhizospheric environment, *P.putida* has demonstrated the capability for successful integration and proliferation in microbial assemblages when employed as bio-augmentation for bioremediation of aromatics (Nancharaiah et al. 2008; Venkata Mohan et al. 2009; Yu et al. 2010). These findings highlight remarkable capability of *P. putida* to compete with natural flora and establish itself in a new environment. Therefore, assessing the role played by competition-driven enhanced siderophore secretion in the establishment of *P.putida* within pre-existing microbial consortia becomes crucial. To understand the role of siderophores in the establishment and proliferation of *P.putida* in an aromatic-rich environment containing other natural flora, we inoculated 10^6^ cells of *P.putida* (WT) and 10^6^ cells of *P.putida* (ΔPpsD) into homogenized activated sludge biomass and soil from the roots consisting of multispecies microbial consortia. As illustrated in Fig. 5,^12^ there was a significantly higher fitness of *P.putida* (WT) over its siderophore mutant [*P.putida* (ΔPpsD)] when grown alongside multispecies consortia with benzyl alcohol as the sole source of carbon. However, the difference in fitness was significantly reduced when the identical experiment was performed in the presence of glucose as the sole carbon source. This observation reemphasizes the crucial role of siderophores in establishing *P.putida* in aromatic-rich, competitive microenvironments.

### Competition effect at gene expression level

**Fig. 6:**
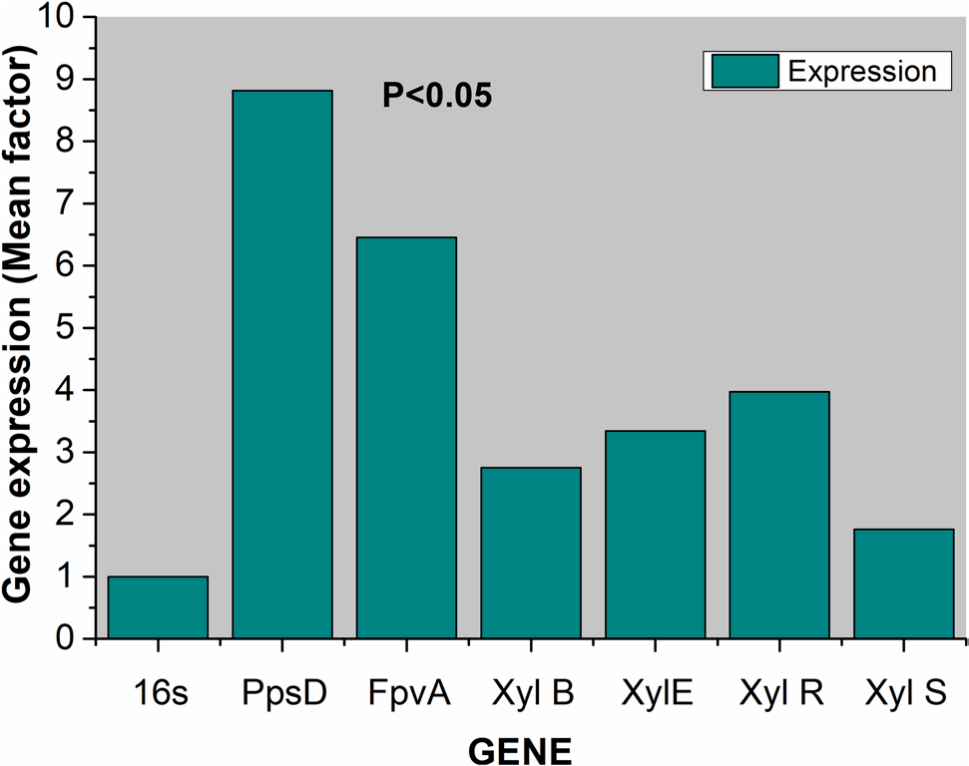
Competition and siderophore secretion at gene expression level: Comparative gene expression profiling of gene associated with aromatic utilization and siderophore secretion in challenged and non-challenged *P.putida*

To provide molecular-level evidence for the role of siderophores in the competition-driven enhancement of siderophore-mediated benzyl alcohol (BA) utilization, we conducted comparative expression profiling of genes involved in BA utilization and siderophore secretion. As shown in Fig. 6,^13^ there was a significant increase in the expression of genes associated with BA utilization (TOL pathway-associated genes) and siderophore synthesis in challenged *P.putida* compared to *P.putida* grown alone. The up regulation of XylR (master regulator of TOL pathways) and XylE (catechol 2, 3-oxygenase), responsible for ring opening reactions, signifies an enhancement in BA utilization in response to competition. Notably, the highest expression levels were observed for PpsD (siderophore synthetase) and FpvA (siderophore receptor), reaffirming the pivotal role played by siderophores in competitor-driven accelerated BA utilization. However, the molecular linkage between genes responsible for siderophore secretion and uptake (ppsD and fpvA) and genes associated with aromatic utilization (TOL pathway) needs further evaluation. Our observations suggest synchronization in the expression of these genes, hinting at the possibility of an existing regulatory network controlling them.

## Discussion

Competition and cooperation are two fundamental social traits of microbes that govern the ecological and evolutionary dynamics of species (McGrath 2024). Iron, one of the most abundant elements on Earth, becomes a focal point of intense competition among microbes due to its limited bioavailability at neutral pH. When multiple organisms secrete siderophores, the competition for iron among these siderophore-producing organisms further intensifies. Consequently, the competitive success of a given siderophore-secreting organism is largely determined by its ability to monopolize siderophore availability and regulate its secretion in response to the competitive environment (Niehus et al. 2017b). The rhizosphere represents a chemically complex environment where dynamic interactions between plants and microbes, as well as among microbes themselves, foster overall plant health (Chepsergon and Moleleki 2023). It has been demonstrated that these interactions are significantly influenced by root secretion, thereby influencing the microbiome of the rhizosphere (Chaparro et al. 2013). For example, It is well documented that under iron starvation, plants secrete higher levels of phenolic (aromatics), leading to a shift in microbial communities towards aromatic-utilizing organisms (Jin et al. 2007b, 2008, 2010b; Tato et al., 2013; Verbon et al., 2017). *Pseudomonas putida* is a prominent member of this community, possessing robust aromatic degradation capabilities. However, the TOL pathway responsible for its aromatic metabolism relies on iron-dependent oxygenases, creating a paradoxical situation. Iron starvation increases the availability of aromatics, but their utilization *by P.putida* requires even more iron. Adding to this complexity, *P.putida* KT2440 produces only one type of siderophore, pyoverdine, and cannot utilize heterologous siderophores. On the contrary, other microbes dominated in this iron limited condition empowered with robust siderophore mediated iron acquisition system (Gu et al. 2020). This raises the intriguing question: how does *P.putida* maintain competitive fitness in this iron-limited environment? One potential mechanism to overcome this limitation is the increased secretion of siderophores in response to the availability of aromatic compounds and the presence of competitors. phenotypic plasticity in siderophore secretion, particularly in response to competition, has been shown to play a critical role in an organism’s competitive success (Leinweber et al. 2018). Beyond iron acquisition, siderophores are known to serve multiple essential functions, including protection against oxidative stress, metal chelation, mitigation of metal toxicity, acting as signaling molecules, enhancing organismal pathogenicity, and notably, functioning as antibiotics or antimicrobial agents (Johnstone and Nolan 2015). As a result, secreting its own siderophore confers significant advantages to an organism, compared to utilizing heterologous siderophores produced by others. For instance, Sexton et al. demonstrated that secreting its own siderophore as an antagonist molecule—beyond iron scavenging alone—yields a more favorable competitive outcome compared to relying solely on heterologous siderophore utilization (Sexton et al. 2017). Pyoverdine, the sole siderophore produced by Pseudomonas *putida*, is well-documented for its strong antimicrobial properties (Boopathi and Rao 1999; Daura-Pich et al. 2020). Consequently, competition-driven enhance siderophore secretion by *P.putida*, not only increases iron assimilation but also provides additional competitive advantages by inhibiting the growth of competing organisms. Enhanced secretion of its own siderophore may compensate for, or even provide advantages over, utilizing heterologous siderophores for competitive success.

**Fig. 7:**
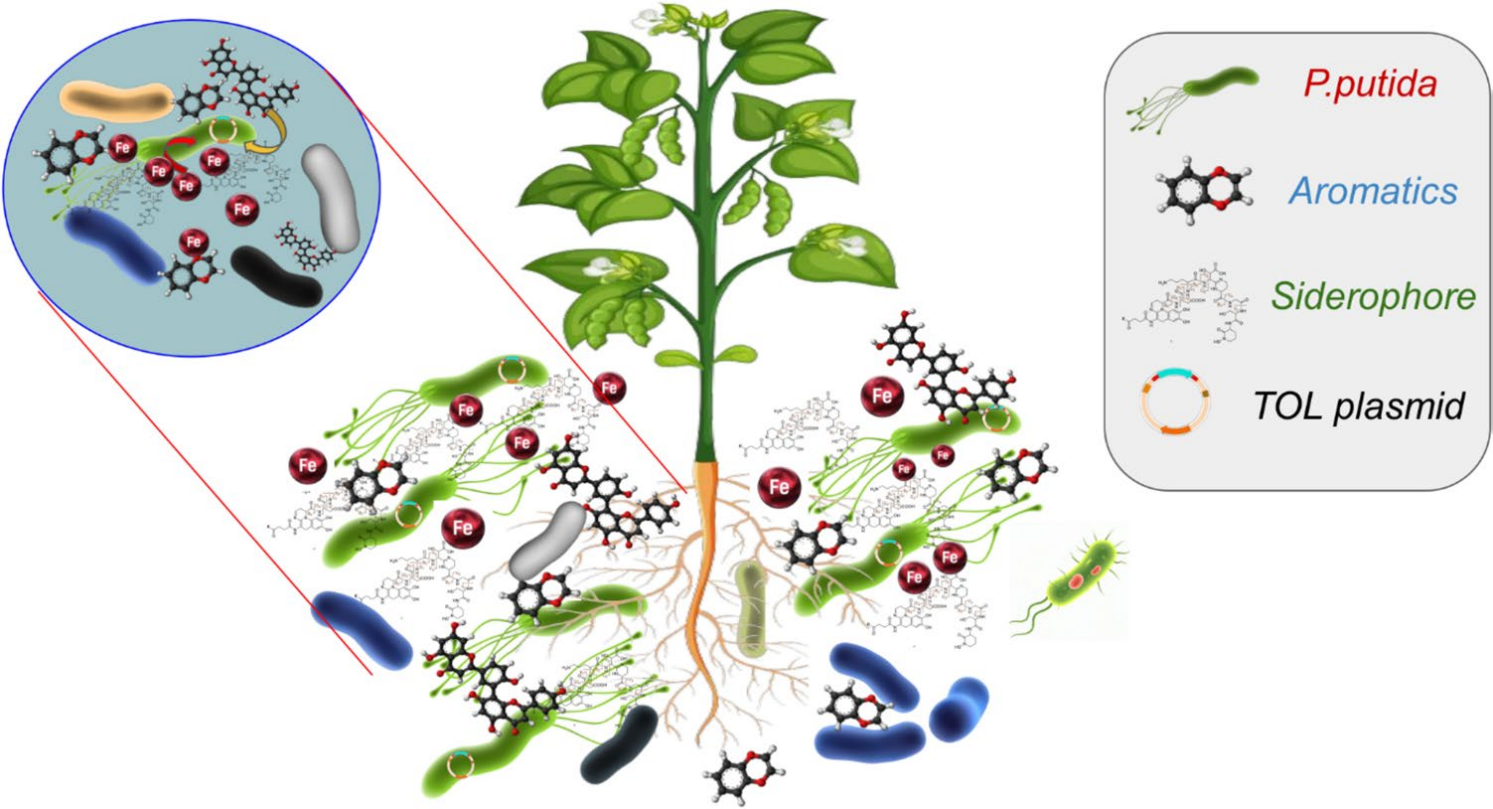
The hypothesis: Schematic representation of hypothesis on adaptive phenotypic plasticity of siderophore secretion in *Pseudomonas putida* providing competitive fitness in iron-limited, aromatic-rich, rhizospheric environment

Production of siderophore or any other metabolites requires significant energy expenditure, and therefore, any unwanted secretion may negatively impact the overall fitness of an organism (O’Brien et al. 2023b). Therefore, fine-tuning the secretion of siderophores based on necessity is a logical strategy for organisms to maximize their benefits. In this study, we demonstrate that the secretion of siderophores by *Pseudomonas putida* is tightly regulated through two layers of control. We observed a marked increase in siderophore production when there was competition for aromatic compounds that required additional iron. However, a similar enhancement in siderophore secretion was not observed when P. putida competed for simpler carbon sources, such as glucose, which rely on iron-independent metabolism. This observation further supports our hypothesis that competition driven enhancement of siderophore secretion by *P.putida* provides fitness advantages in an aromatic-rich environment. Figure 7,^14^ provides a schematic representation of our hypothesis on competition driven enhanced siderophore secretion conferring the fitness advantage to *P.putida* in the aromatic-rich, rhizospheric environment.

In addition to its critical role as a plant growth-promoting organism, *Pseudomonas putida* has garnered significant attention from researchers for its remarkable capabilities in utilizing aromatic compounds. *P.putida*’s ability to efficiently metabolize aromatic compounds positions it as a promising candidate for various environmental and industrial processes, particularly in the context of oil spill remediation and the biodegradation of aromatic pollutants. (Nancharaiah et al. 2008b; Venkata Mohan et al. 2009b; Xu et al. 2018; Aziz et al. 2024). It is well documented that most bacteria failed to perform equivalent tasks in a natural environment, which was otherwise exceptionally executed in a laboratory environment. One of the primary reasons behind this failure is attributed to microbial competition (Abtahi et al. 2020; Aziz et al. 2024). However, *P.putida* has been shown to be successfully integrated (bioaugmentation) in polymicrobial activated sludge and aerobic granules, clearly establishing their competitive fitness (Nancharaiah et al. 2008b; Venkata Mohan et al. 2009b).

Moreover, beyond aromatics utilization, numerous reports highlight the central role of siderophores in the overall survival and proliferation of *P.putida* (Dinkla et al. 2001b; Molina et al. 2005, 2006; Matilla et al. 2007b)*.* Consequently, the accelerated secretion of siderophores will have a multidimensional positive impact on the competitive survival and proliferation of *P.putida* in such natural environments.

## Conclusion

The *Pseudomonas* group of organisms stands out as a dominant community in diverse ecological niches, underscoring their exceptional survival capabilities and adaptability in the face of ever-changing environmental conditions. Despite their widespread presence, there is a notable dearth of information on the physiological determinants contributing to their competitiveness and adaptability. Our findings, shedding light on competitor-driven enhanced siderophore secretion, offer insight into one of these potential attributes that enhance the survival and proliferation of *P.putida* in iron-restricted, aromatic-rich environments. Moreover, our observations underscore an alternative mechanism by which the organism compensates for its limitations concerning exploiting a narrow spectrum of nutrients. While we acknowledge the absence of experimental evidence regarding the molecular mechanisms behind competitor-driven enhanced siderophore secretion in *P.putida,* we firmly believe that our initial insights open avenues for exploring the often-overlooked realm of competitor sensing and its profound impact on microbial survival and proliferation.

## Supplementary Information

Below is the link to the electronic supplementary material.

Supplementary file1 (TIF 230 KB)

Supplementary file2 (TIF 7923 KB)

Supplementary file3 (TIF 1183 KB)

## Data Availability

No datasets were generated or analysed during the current study.

## Footnotes

1 Figure 1A

2 Figure 1B

3 Figure 2A

4 Figure 2B

5 Figure S1.

6 Figure 3A

7 Figure 3B

8 Figure 3C

9 Figure S2.

10 Figure 4

11 Figure S3.

12 Figure 5

13 Figure 6

14 Figure 7
