# Supplementary figures and images for: Competition-driven phenotypic plasticity in Iron acquisition and aromatic utilization confers a fitness advantage to Pseudomonas putida in an Iron-limited rhizospheric environment
Source: World J Microbiol Biotechnol. 2024 Nov 20;40(12):386. doi: 10.1007/s11274-024-04192-8 (PMC11579168; doi:10.1007/s11274-024-04192-8)

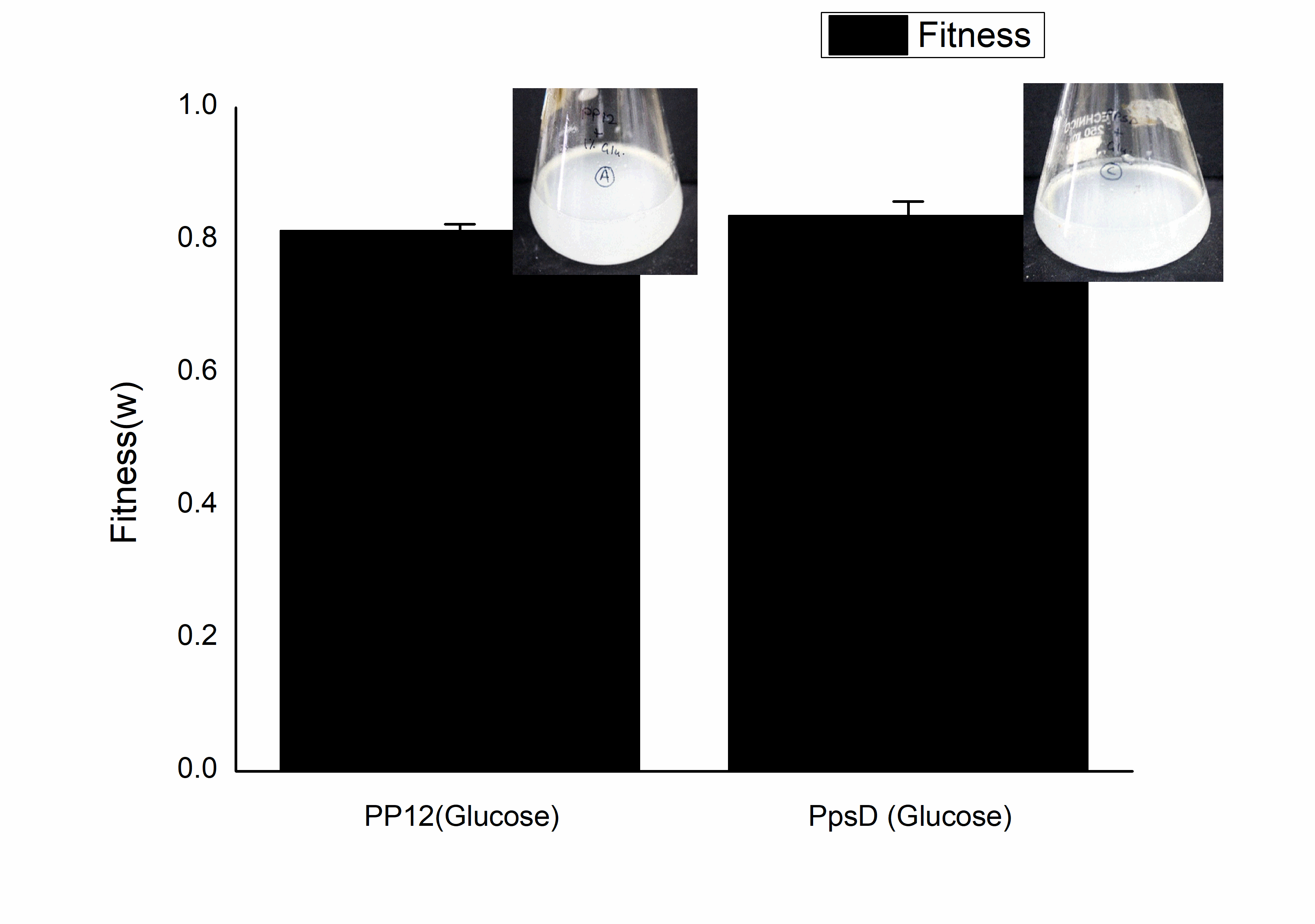

Supplement: Supplementary file 3 — Supplementary file3 (TIF 1183 KB) [file 11274_2024_4192_MOESM3_ESM.tif]
